# Retraction notice to “Insights into chemistry, extraction and industrial application of lemon grass essential oil -A review of recent advances” [Food Chemistry: X 22 (2024) 101521]

**DOI:** 10.1016/j.fochx.2026.103769

**Published:** 2026-03-30

**Authors:** Barjees Ashaq, Khansa Rasool, Samira Habib, Iqra Bashir, Naseh Nisar, Sehrish Mustafa, Qudsiya Ayaz, Gulzar Ahmad Nayik, Jalal Uddin, Seema Ramniwas, Robert Mugabi, Sajad Mohd Wani

**Affiliations:** a Division of Food Science and Technology, Sher-e-Kashmir University of Agricultural Sciences and Technology, Kashmir, 190025, J&K, India; b Department of Food Science & Technology, Govt. Degree College, Shopian 192303, J&K, India; c Department of Pharmaceutical Chemistry, College of Pharmacy, King Khalid University, Asir 61421, Saudi Arabia; d University Centre for Research and Development, Chandigarh University, Gharuan, Mohali 140413, Punjab, India; e Department of Food Technology and Nutrition, Makerere University, Kampala, Uganda

This article has been retracted: please see Elsevier policy on Article Correction, Retraction and Removal (https://www.elsevier.com/about/policies-and-standards/article-withdrawal).

This article has been retracted at the request of the Editor-in-Chief.

The authors have plagiarized figures and some text that had already appeared in a book chapter: Mukarram, M., Khan, M.M.A., Zehra, A., Choudhary, S., Naeem, M., Aftab, T. (2021). Biosynthesis of Lemongrass Essential Oil and the Underlying Mechanism for Its Insecticidal Activity. In: Aftab, T., Hakeem, K.R. (eds) Medicinal and Aromatic Plants.

One of the conditions of submission of a paper for publication is that authors declare explicitly that their work is original and has not appeared in a publication elsewhere. Re-use of any data should be appropriately cited. Apologies are offered to readers of the journal that this was not detected during the submission process.

